# Addition of High Molecular Weight Hyaluronic Acid to Fibroblast-Like Stromal Cells Modulates Endogenous Hyaluronic Acid Metabolism and Enhances Proteolytic Processing and Secretion of Versican

**DOI:** 10.3390/cells9071681

**Published:** 2020-07-13

**Authors:** Jiapeng Xue, Jinnan Chen, Quan Shen, Deva Chan, Jun Li, Adam P. Tanguay, Tannin A. Schmidt, Faizan Niazi, Anna Plaas

**Affiliations:** 1 Department of Internal Medicine (Division of Rheumatology), Rush University Medical Center, Chicago, IL 60612, USA; jiapengxue2889@hotmail.com (J.X.); chenjinnan001@gmail.com (J.C.); jun_li@rush.edu (J.L.); 2 Department of Neurosurgery, Rush University Medical Center, Chicago, IL 60612, USA; eric.quanshen@gmail.com; 3 Department of Biomedical Engineering, Rensselaer Polytechnic Institute, Troy, NY 12180, USA; deva.chan.phd@gmail.com; 4 Biomedical Engineering Department, University of Connecticut Health Center, Farmington, CT 06030, USA; atanguay@uchc.edu (A.P.T.); tschmidt@uchc.edu (T.A.S.); 5 Ferring Pharmaceuticals Inc., Parsippany, NJ 07054, USA; Faizan.Niazi@ferring.com

**Keywords:** osteoarthritis, synovium, stromal cells, aggrecan, versican, PRG4, microvesicles, mesenchymal progenitors, hyaluronic acid therapeutics

## Abstract

We have examined the effect of exogenous linear chain high molecular weight hyaluronic acid (HMW HA) on endogenously synthesized hyaluronic acid (HA) and associated binding proteins in primary cultures of fibroblast-like stromal cells that were obtained by collagenase digestion of the murine peripatellar fat pad. The cultures were expanded in DMEM that was supplemented with fetal bovine serum and basic fibroblast growth factor (bFGF) then exposed to macrophage-colony-stimulating factor (MCSF) to induce macrophage properties, before activation of inflammatory pathways using *E. coli* lipopolysaccharide (LPS). Under all culture conditions, a significant amount of endogenously synthesized HA localized in LAMP1-positive lysosomal vesicles. However, this intracellular pool was depleted after the addition of exogenous HMW HA and was accompanied by enhanced proteolytic processing and secretion of de novo synthesized versican, much of which was associated with endosomal compartments. No changes were detected in synthesis, secretion, or proteolytic processing of aggrecan or lubricin (PRG4). The addition of HMW HA also modulated a range of LPS-affected genes in the TLR signaling and phagocytosis pathways, as well as endogenous HA metabolism genes, such as *Has1*, *Hyal1*, *Hyal2*, and *Tmem2*. However, there was no evidence for association of endogenous or exogenous HMW HA with cell surface CD44, TLR2 or TLR4 protein, suggesting that its physiochemical effects on pericelluar pH and/or ionic strength might be the primary modulators of signal transduction and vesicular trafficking by this cell type. We discuss the implications of these findings in terms of a potential in vivo effect of therapeutically applied HMW HA on the modification of osteoarthritis-related joint pathologies, such as pro-inflammatory and degradative responses of multipotent mesenchymal cells residing in the synovial membrane, the underlying adipose tissue, and the articular cartilage surface.

## 1. Introduction

Many chronic musculoskeletal diseases are driven by innate inflammatory responses following acute or chronic soft tissue injuries and they can lead to tissue destruction and pain with long term disabilities. The majority of research on these diseases, such as osteoarthritis (OA) [1,2,3] and the tendinopathies [4,5], have focused on defining the involvement of specific tissue and cell types as well as inflammatory mediators and down-stream signaling pathways. These considerations have largely guided translational research approaches in the attempt to discover new therapeutics to slow disease progression and to alleviate pain and disability.

Research on the pathogenesis of chronic diseases in organs such as the liver, gut, kidney, respiratory, and cardiovascular tissues, has evaluated the prominent role of the polysaccharide-rich pericellular matrix (glycocalyx) [6,7,8]. However, this has been studied in a more limited way in the musculoskeletal diseases.

Of particular interest in this area is hyaluronic acid (HA) and its binding proteins, versican (VCAN), pentraxin 3 (PTX3) [9,10], heavy chains (HC) 1 and 2 of the inter-alpha-trypsin inhibitor [11,12,13], and aggrecan (ACAN). In addition to being present in inflamed regions of organs and connective tissues, these complexes have also been found to be associated with cells in the innate immune system (in particular macrophages) or pluripotent progenitor cells involved in healing responses of injured connective tissues. It is also notable that interfering with the inflammatory HA matrix has been an effective approach in restoring the function of lining cells in respiratory diseases such as cystic fibrosis and chronic obstructive pulmonary disease (COPD) [14,15]. While a number of studies report on the effects of (HMW HA) injections in diminishing tissue inflammation and joint pain in symptomatic OA patients, the lack of appropriate global guidelines for clinical trial designs has resulted in heterogeneity of the research, thus impeding the more widespread use of this therapy in slowing the progression of OA [16,17].

Much of the mechanistic research of intra-articular (IA) HMW HA injections has focused on the potential action as a lubricant, while only a few basic research studies have examined the in vivo biological effects on cells in the target tissues [18,19,20,21]. These studies report that, in addition to affecting neuronal pathways, cells in the synovial lining and underlying adipose tissue, as well as the periosteal/perichondrial cell populations, can be affected. Moreover, in vitro cell culture studies with synovium derived fibroblasts or macrophage cell lines [22,23,24,25,26] have focused on examining the effect of exogenous HMW HA on the modulation of NFkB-mediated gene expression, but none of these studies included an assessment on endogenous HA metabolism.

The objective of the present study was to examine the effect of exogenously added linear chain HMW HA on the synthesis, secretion, and processing of endogenous HA and associated binding proteins, on fibroblastic stromal cells. These were obtained by collagenase digestion of the murine peripatellar fat pad and established as monolayers in basic fibroblast growth factor (bFGF) supplemented media. Cellular responses to HMW HA were examined with and without exposing the cells to macrophage-colony-stimulating factor (MCSF) [27,28,29]. Furthermore, to mimic the activation of OA-related Toll-like receptor (TLR) signaling events [30,31], we used *E. coli* Lipopolysaccharide. This signaling converges with the Nfkb pathway, as do multiple cytokines often employed in the context of OA inflammation such as IL1β (Interleukin 1 beta), IL6 (Interleukin 6), and TNF-α (Tumor Necrosis Factor alpha). Furthermore, the endotoxin has been used as an inflammatory agent in a number of synovitis-driven OA animal models [32,33]. The data are discussed in terms of a potential in vivo effect of therapeutic HMW HA on inflammatory wound-healing responses of osteoarthritic joint tissues [34], and the possible responses by multipotent mesenchymal cells, known to reside in the synovial membrane, the underlying adipose tissue, and the articular cartilage surface.

## 2. Materials and Methods

### 2.1. (Fibroblast-Like-Stromal Cell) FLSC Cultures

All of the mouse experiments were carried out under an Institutional IACUC approved protocol (17-019). Ten to twelve week old C57Bl6 male mice were sacrificed and the peripatellar fat pad, including adherent the synovial membrane, was removed immediately and then placed into sterile CO_2_-independent medium on ice [35]. Tissues from twelve mice were pooled for each separate cell preparation. Following a brief wash with ice-cold PBS, the pooled tissue was incubated in 4 mL CO_2_ independent medium (LifeScience Thermo Fisher, Leawood, KS, USA) containing 20 mg of Collagenase II (Roche), and digested for 1 h at 37 °C. Tissue remnants were further dispersed by pipetting with a 1 mL pipettor and the cells were separated from the collagenase solution by centrifugation at 900 *g* for 15 min. Cell pellets were washed once with 5 mL PBS before suspension in DMEM, with 5 mM glucose, 1 mM glutamine, and 10% FBS (Atlanta Biologics, Flowery Branch, GA, USA, 30542) and 2 ng/mL bFGF (HuR, R&D Systems Minneapolis, MN), required to enhance proliferation and viability of cells at the low plating densities of ~1.5 × 10^3^ cells per well in 12-well plates (Falcon). Non-adherent cells were removed after 24 h and culture medium changed every 48 h for 6–8 days until ~70–80% confluency. The cultures were then treated with either 2 ng/mL bFGF or 5 ng/mL murine MCSF (MuR; PepProtech, Cranbury, NJ, USA) to enhance macrophage properties for 36 h (see Supplementary Materials Figure S1).

### 2.2. Treatment of Cultures with LPS and HMW HA

The medium was removed and replaced with fresh medium, supplemented (or not) with 1 μg/mL LPS from *E. coli* O111:B4 (Millipore Sigma L4391) to stimulate pro-inflammatory pathways through TLR4, which was abundantly expressed in these cultures (Figure S3). After 4 h incubation, media were removed and replaced with fresh medium (DMEM with 5 mM glucose, 1 mM glutamine and 10% FBS) supplemented with bFGF or MCSF in the absence or presence of 100 μg/mL low endotoxin HMW HA (Euflexxa^®^ Lots: L14858A; M14127A; N11705A; obtained from Ferring Pharmaceuticals, Inc., Parsippany, NJ, USA). The media were removed and stored frozen at −20 °C until further analyses (see Figure S1 for experimental timeline). None of the three Euflexxa^®^ preparations showed endotoxin responses in the, cultures as assessed by the lack of *Nos2*, *Il6*, and *Tnf*α induction. Furthermore, they all resulted in the effective depletion of intracellular HA assessed by confocal microscopy (data not shown).

### 2.3. RTqPCR Assays

Cells (~1.2 × 10^6^) from three combined wells were solubilized in 1.5 mL TRIzol^®^, and RNA isolated with the RNAeasy kit (Qiagen, Hilden, Germany), as described [35]. cDNA was synthesized with the First-Strand Synthesis Kit (Invitrogen, Carlsbad, CA, USA). RTqPCR was performed with the Taqman™ platform (LifeScience Technologies) assaying genes for multipotency phenotype (*Col1a1*, Collagen Type 1 alpha 1; *Col3a1*, Collagen Type 3 alpha 1; *Col5a1*, Collagen Type 5 alpha 1; *Cd34*, CD34 Molecule; *Gfap*, Glial Fibrillary Acidic Protein; *Ncam1*, Neural Cell Adhesion Molecule 1; *Pparg*, Peroxisome proliferator-activated receptor gamma), macrophage phenotype (*Cd163*, CD163 Molecule; *Emr1*, EGF module-containing mucin-like receptor; *Itgam*, Integrin Subunit Alpha M), HA metabolism (*Has1*, Hyaluronan Synthase 1; *Has2*, Hyaluronan Synthase 2; *Cd44*, CD44 Molecule; *Ptex3*, Pentraxin 3; *Tnfaip6*, TSG6 (TNF-inducible gene 6 protein); *Hyal1*, Hyaluronidase 1; *Hyal2*, Hyaluronidase 2; *Cemip*, Cell Migration Inducing Hyaluronidase; *Tmem2*, Cell Surface Hyaluronidase), LPS-sensitive genes (*Il6*, Interleukin 6; *Nos*2, Nitric Oxide Synthase 2) and ECM molecules (*Acan*, Aggrecan; *Vcan*, Versican; *Prg4*, Lubricin). All of the protein, gene and primer details are given in Supplementary Materials Table S1a. The samples were also assayed in TaqMan^®^ Arrays (Fisher LifeScience Technologies) for Mouse Toll-Like Receptor Signaling Genes (RA47VTN) and Mouse Phagocytosis Pathway Genes (RAXGPXN) (see Table S1b,c).

Changes in transcript abundance (ΔCt = Ct for transcript of interest minus Ct for the housekeeping gene, *B2m*) were used to calculate ΔΔCts of treated vs. non-treated groups and then used to determine the fold changes as 2^−ΔΔCt^. Of note, three additional housekeeping genes (*Gapdh*, *Actb*, *and Gusb*) were included in all assays, with only *B2m* demonstrating minimal variation in Ct values across experimental samples assayed. A one-way ANOVA with Tukey’s post-hoc test was conducted using GraphPad Prism 5 (La Jolla, CA, USA) on the ΔCt values to determine the significance (*p* < 0.05) in modulated expression of genes after treatment with LPS or HMW HA), as compared to no additives. An unpaired Student’s *t*-test was used to compare bFGF and MCSF values for each experimental group.

### 2.4. Confocal Microscopy

The cells were cultured as described above, but on glass coverslips in twelve well plates. Pretreatment of the coverslips to obtain cell adherence and spreading equivalent to that seen on plastic surfaces, and to lower auto-fluorescent background, was carried out by consecutive washes in 0.5% (*v*/*v*) formic acid, water and 70% (*v*/*v*) ethanol. Media were removed and attached cells washed briefly with ice-cold PBS before fixation and storage at 4 °C in Histochoice™ containing 10% absolute ethanol and stored at 4 °C until further processing. To remove extracellular HA, the cells were incubated with 0.1 mU of *Streptomyces* Hyaluronidase (S Hyase) (Sigma Aldrich, St. Louis, MO, USA) in PBS containing Complete Mini protease inhibitors (Roche (Basel, Switzerland) with 5 mM EDTA for 10 min. at room temperature. The digest was removed and cells fixed and stored as above. Prior to immunostaining, the fixative was removed and the coverslips washed for 2 min. in ice-cold methanol. Staining was performed essentially as previously described [36] after blocking with donkey serum (5 μg/mL in PBS). Primary antibodies were: anti-CD44 (ab189524 Abcam, Cambridge, MA, USA; 1:250); anti-DPE, Figure S3 1 μg/mL), anti-DLS/CDAG (Figure S3 1 μg/mL each), anti-LAMP1 (Lysosomal-associated membrane protein1) (ab208943 Abcam; 1:100), anti-Calnexin (ab213243 Abcam, 0.5 μg/mL), and anti-EEA1 (Early Endosomal Marker Protein) (ab2900, Abcam, 1 μg/mL). Alexa Fluor 568 donkey anti-rabbit IgG (A10047, Invitrogen, 4 μg/mL) was used as secondary antibody. Protein IHC was followed by incubation with biotinylated HA-binding protein (bHABP) (MilliporeSigma Burlington, MA, USA) diluted at 0.5 μg/mL in PBS) for 2 h followed by incubation with Alexa Fluor488 Streptavidin (S11223, Thermo Fisher, 4 μg/mL). All of the samples were counterstained with DAPI (Sigma Aldrich, 1 mg/mL in PBS), and then cover-slipped with ProLong™ Anti-Fade Reagent (ThermoFisher Invitrogen). Confocal imaging was performed using a Zeiss LSM710 Confocal microscope fitted with a 63X1.46NA oil immersion lens.

### 2.5. Solubilization of Cell Layer Associated ACAN, VCAN, and PRG4

Following 16 h of incubation in the absence of presence of 100 μg/mL HMW HA, media were removed and cell layers briefly washed with ice-cold serum free CO_2_-Independent medium. Fifty microliters of PBS/50 mM ammonium acetate (pH 7.5) containing 2× protease inhibitors (Complete Mini; Roche with 5 mM EDTA) with 20 mU chondroitinase ABC (AmsBio) was added to each well, incubated for 30 min. at 37 °C followed by addition of 60 μL 2× Sample Buffer™ (BioRad, Hercules, CA, USA) containing 1 mM DTT. Solubilized cell layers were recovered into Eppendorf tubes and heated at 100 °C for 10 min. The samples were stored at −20 °C before electrophoresis.

### 2.6. Purification of Aggrecan, Versican and PRG4 from Culture Medium

This was carried out, as described previously [35,37]. Briefly, the conditioned media were adjusted to 7 M urea, 15 mM benzamidine, 5 mM EDTA, 1 mM AEBSF, 5 mM iodoacetamide, 5 μg/mL pepstatin, 10 μg/mL leupeptin and pH 8.0, and clarified by centrifugation (14,000× *g* for 5 min.). The supernatants were fractionated by anion exchange chromatography while using DE52 resins. High salt fractions were dialyzed against multiple changes of distilled water, each sample divided into two portions, one for VCAN and one for ACAN western blotting, speedvac dried, dissolved in 60 μL Sample Buffer (BioRad) containing 1 mM dithiothreitol, heated at 100 °C for 10 min. and then stored at −20 °C until SDS PAGE.

### 2.7. Western Blotting

SDS PAGE on Novex™ 4–12% Tris-Glycine Mini Gels (Invitrogen) and Western blotting was performed, as previously described [35,37]. Briefly, following electro transfer, the membranes were incubated with 1 μg/mL anti-DPE or a mixture of anti-CDAG/anti-DLS, each at 1 μg/mL (see Supplementary Materials Figure S3 for location of the epitopes), and developed using the Pierce ECLPlus Kit (Thermo Fisher Scientific, Waltham, MA, USA). Reprobes with anti-PRG4 (MAb 9G3, 0.05 µg/mL; Millipore Sigma, Burlington, MA, USA) [37,38] were performed by washing membranes three times for ten minutes each with 8 mL of TBS containing 0.1% Tween 20 (Bio-Rad, Hercules, CA, USA). The membranes were incubated with and were developed using the SuperSignal West Femto Maximum Sensitivity Substrate (Thermo Scientific, Waltham, MA, USA). To confirm equivalent loading amounts, western membranes from media samples and cell extracts were reprobed with anti-HC1 (1 μg/mL) [30] or anti-β-actin (1:5000 MAbAC-15; Novus Biologicals, Littleton, CO, USA). Imaging was performed with GBox Chemi-XX9 (Syngene, Frederick, MD, USA).

## 3. Results

### 3.1. HA Metabolism in FLSC Cultures Expanded in bFGF and Treated with MCSF to Enhance a Macrophage Phenotype

**Figure 1:**
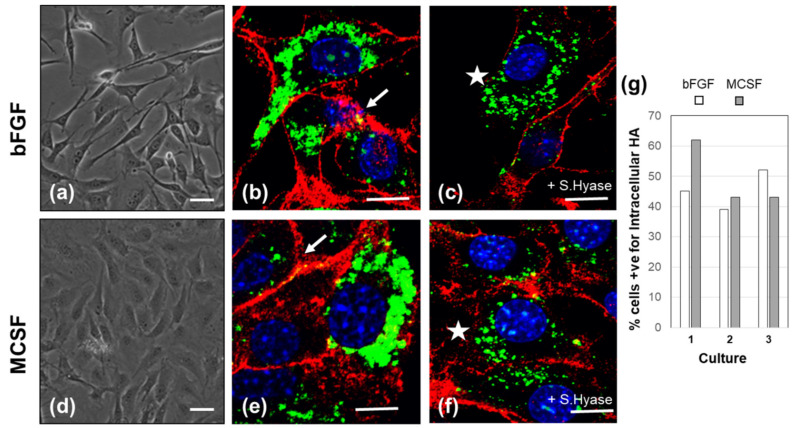
Morphological appearance and endogenous HA localization of basic fibroblast growth factor (bFGF) and macrophage-colony-stimulating factor (MCSF) treated FLSC cultures. Phase contrast images (**a**,**d**) Confocal localization of HA (green fluorescence) and CD44 (red fluorescence) (**b**,**c**,**e**,**f**). Panels (**c**,**f**) show images of cells which were digested with *S.* Hyase prior to fixation, to remove extracellular HA, revealing abundant intracellular HA (marked with white * in panels **c**,**f**). Panel **g** shows the % of cells containing intracellular HA from a total of *n* = 50–70 cells, in images taken from 5 separate areas of coverslips prepared from 3 individual cultures. Cell fixation, and staining protocols for HA (with bHABP) and CD44 (with anti-CD44) were carried out as described in the Methods Section. Space bar = 10 μm.

Following adherence to the culture well, the cells proliferated rapidly in bFGF-supplemented medium, and reached 70–80% confluency after seven days (Figure 1a). The exposure of the cells to MCSF for 36 h caused no marked proliferation, but the cells acquired a more flattened morphology (Figure 1d). Furthermore, it resulted in a decreased expression of mesenchymal gene, *Col1a1*, and increased expression of macrophage-characteristic genes *Emr1*, and *Cd163* (Figure S2). We assayed the expression of genes involved in HA synthesis and extracellular organization previously reported to be expressed by fibroblastic cell types (*Has1*, *Has2*, *Tnfaip6*, *Petx3*, and *CD44*) and HA degradation (*Hyal1*, *Hyal2*, *Tmem2*, and *Cemip*) to determine the capacity of the cells for endogenous HA metabolism. All of the genes showed robust expression levels, in both bFGF and MCSF supplemented cultures, and there was no detectable difference between the two growth factors. Furthermore, expression remained essentially constant for 4 to 36 h after the addition of these factors (Table 1).

The cells were stained with bHABP to localize HA and co-stained with anti-CD44 to demarcate the cell surface (Figure 1b,e, bFGF or MCSF, respectively). With either factor, HA was found distributed over the cell body, but there was no detectable staining of the ECM. Furthermore, very limited co-localization of HA with CD44 was detected (white arrow heads, Figure 1). When the cells were pretreated with *S.* Hyase before fixation and dual IHC, a robust cell-associated HA staining remained, indicating that a large portion of the HA produced was located inside the cells. The pool of intracellular HA was likely the product of HAS2, since cultures established from *Has1*−/− *Has3*−/− mice also contained these HA-rich inclusions (data not shown).

**Figure 2:**
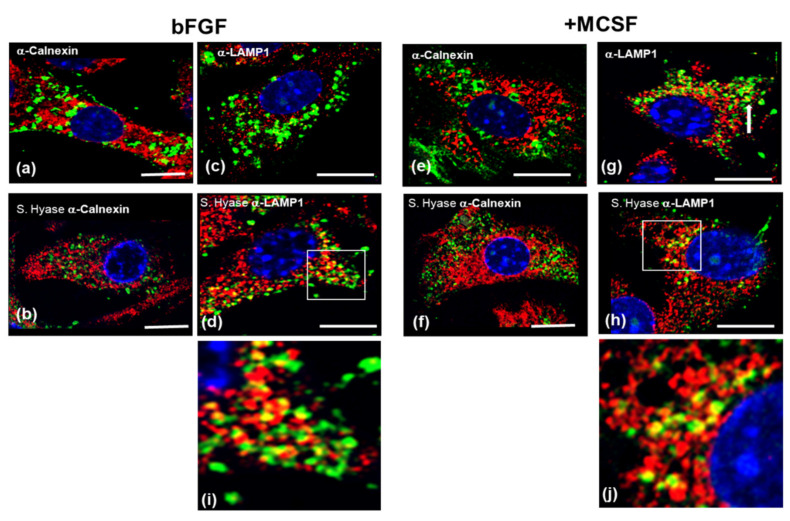
Localization of endogenous intracellular hyaluronic acid (HA) to endoplasmic reticulum (ER) or Lysosomal compartments in bFGF and MCSF treated FLSC cultures prior to treatment with LPS or exogenous high molecular weight hyaluronic acid (HMW HA). Cells were dual labelled with bHABP and either anti-Calnexin (**a**,**b**,**e**,**f**) or anti-LAMP1 (**c**,**d**,**g**,**h**) antibodies without (**a**,**c**,**e**,**g**) or with S Hyaluronidase pretreatment (**b**,**d**,**f**,**h**). Intracellular HA was partially co-localized with anti-LAMP1 (lysosomes, white arrow heads in **d**,**h**). There was no evidence for localization of intracellular HA to the ER compartments. Panels (**i**,**j**) show higher magnifications of the boxed areas in panels (**d**,**h**), respectively to illustrate the co-localization of HA with LAMP1-+ve intracellular vesicles. The bar-graph shows the quantitation of cells positive for intracellular HA, which was determined as described in the Methods Section. Space bars = 10 μm.

To further delineate the intracellular location of this HA pool [39] to either the ER [40] or lysosomal compartments [41], the cultures were co-stained for HA and antibodies to Calnexin, an endoplasmic reticulum (ER)-specific chaperone protein [42] (Figure 2a,e native; Figure 2b,f + *S.* Hyase treatment) or to LAMP1, a lysosomal marker protein (Figure 2c,g, native; Figure 2d,h + *S.* Hyase treatment). A significant co-localization of HA with LAMP1 was seen, especially after *S.* Hyase pretreatment (white arrows, panels d & h), but it was virtually absent from calnexin positive regions. This indicates that the intracellular pool of HA in these cultures is derived from cell–derived newly synthesized (“endogenous”) HA, likely destined for lysosomal degradation [43].

### 3.2. Effect of LPS on Cell-Associated HA in FLSCs Expanded in bFGF and Treated with MCSF

bFGF and MCSF treated cells were exposed for 4 h to LPS (1 µg/mL), followed by an additional 16 h in complete medium without LPS, in order to examine the effect of a pro-inflammatory stimulus on HA metabolism and localization. Preliminary experiments to determine a dose-response of the cells to the endotoxin showed that the short time exposure to high LPS in complete medium resulted in the maximal response as determined by expression of TLR4-sensitive genes (*Nos2*, *Il6*), and this activation was sustained over 16 h after removal of LPS (Table 2).

As expected, from the multitude of published data on increased HA metabolism in inflammation involving TLR pathway activation and Nfkb signaling, acute LPS treatment also affected the transcript abundance of genes in HA synthesis and ECM assembly (Table 2). Increases during the acute LPS treatment in either bFGF or MCSF cultures were found for *Has1* (~2 fold), *Has2* (~1.6 fold), *Ptx3* (5–12 fold), and *Tnfaip6* (6–12 fold), with no changes in CD44 expression. Further, alterations were observed in the expression in the four hyaluronidases, all of which were decreased in their mRNA levels.

**Figure 3:**
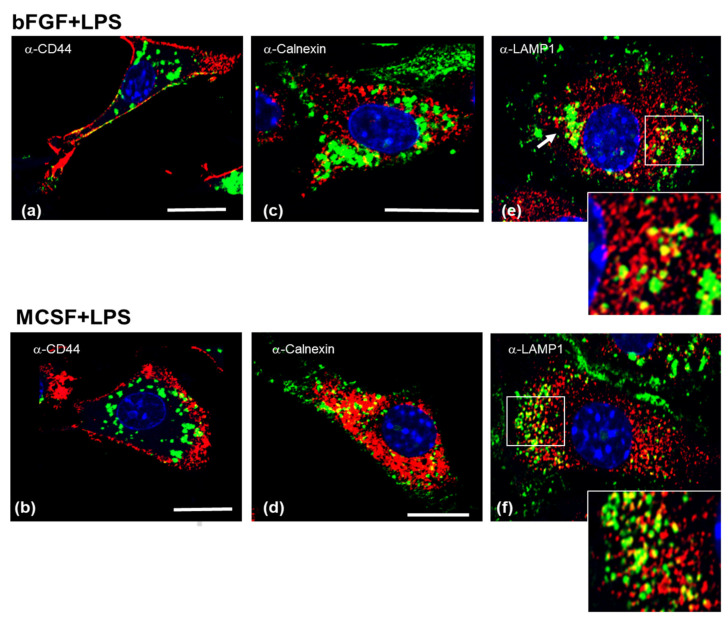
LPS Stimulation has no effect on intracellular localization and distribution of endogenous HA. Cells treated with bFGF (**a**,**c**,**e**) or MCSF (**b**,**d**,**f**), then stimulated with LPS for 4 h, followed by 16 h incubation in basal serum containing medium, as described in the Methods. Fixed cells were then dual labelled with bHABP and either anti-CD44 (**a**,**b**), anti-Calnexin (**c**,**d**) or anti-LAMP1 (**e**,**f**). Panels (**i**,**j**) show higher magnifications of the boxed areas in panels (**d**,**h**), respectively, to illustrate the co-localization of HA with LAMP1-+ve intracellular vesicles. Space bars = 10 μm.

However, after an additional 16 h incubation without LPS, the mRNA levels increased further above pre-LPS levels for *Has1* (~5- and 9-fold) and *Ptx3* (67 and 132 fold), whereas *Tnfaip6* remained at the acute stimulated levels with *CD44* and *Cemip* remaining unchanged from non-LPS levels. Notably, in MCSF supplemented cultures, during the 16h without LPS, the levels of mRNA were significantly decreased for *Has2* (~3 fold), *Hyal1* (~4 fold), *Hyal2* (~9 fold) and *Tmem2* (~3 fold) relative to non-LPS control levels. The 4 h LPS stimulation (data not shown) or incubation for 16 h without LPS (Figure 3) did not alter staining of intracellular HA, or its predominant co-localization with LAMP1 (Figure 3c,d).

It should also be noted that the expected ‘cable’ structures of extracellular HA [10,12] were not observed in association with HCs, PTX3, and VCAN that have been previously reported in smooth muscle cells exposed to pro-inflammatory stimuli, such as poly-IC or high glucose. Although *Tnfaip6* and *Ptx3* gene expression was robustly stimulated by LPS, the FBS preparations used in the current study did not contain intact HCs bound to bikunin-CS, but almost exclusively contained 35-kD fragments of HCs (Figure S4). These are likely thrombin/plasmin generated fragments produced during commercial preparation of the serum and are not substrates for transfer to HA by TSG6 [44].

### 3.3. Effect of Addition of HMW HA on Endogenous HA Metabolism in Basal or LPS Stimulated FLSC Cultures

The HMW HA was added during both the acute 4 h LPS stimulation period or during the 16 h post LPS treatment period, as described in the Methods Section and shown in Figure S1. The assays of gene expression for the TLR4-responsive genes, *IL6* and *Nos2*, as well as the HA metabolism genes, did not show any changes when HMW HA was added together with LPS. In addition, when gene expression was assayed in FLSCs maintained for 4 h in complete medium without LPS, and after addition of HMW HA during the subsequent 16 h, only a small (~2 fold) stimulation of *IL6* was detected, with no changes in mRNA abundance for *Nos2* or any of the HA metabolism genes (Table 3).

In contrast, in cultures pretreated for 4 h with LPS, the addition of HMW HA further increased *IL6* gene expression by ~20 fold (*p* < 0.001) and ~40 fold (*p* < 0.001) for bFGF and MCSF supplemented cultures, respectively, and also decreased the expression of *Nos2* by ~30 fold (*p* < 0.001) in bFGF and ~7 fold (*p* < 0.001) in MCSF. Furthermore, LPS increased mRNA abundance ~2–3 fold for several of the HA metabolism genes, *Has2*, *Hyal1*, *Hyal2*, and *Tmem2* during the post-LPS (16 h) exposure and for both MCSF and bFGF supplemented media.

**Figure 4:**
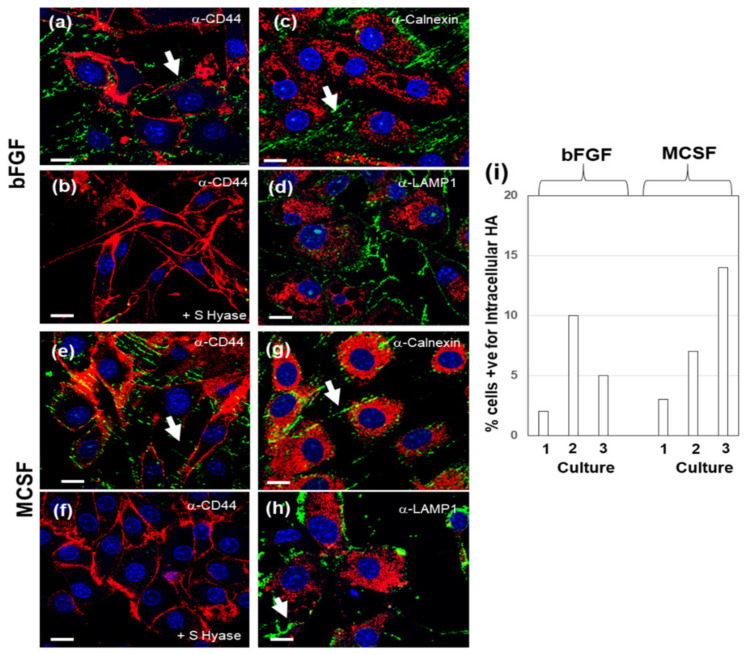
Effect of the addition of exogenous HMW HA on association of endogenous HA with CD44, Calnexin and LAMP1. Cells treated with bFGF (**a**–**d**) or MCSF (**e**–**h**) and incubated for 16 h in basal FBS containing medium with 100 μg/mL HMW HA. Fixed cells were then dual labelled with bHABP and either anti-CD44 (**a**,**e**), anti-Calnexin (**c**,**g**), or anti-LAMP1 (**d**,**h**). Additional cells were pretreated with S Hyaluronidase prior to fixation, to remove extracellular prior to staining with bHAPB and anti-CD44 (**b**,**f**). The bar-graph in panel (**i**) shows the quantitation of cells positive for intracellular HA, which was determined as described in the Methods Section. Space bars = 10 μm. White arrowheads indicate the deposition of extracellular HA in between cell groups.

**Figure 5:**
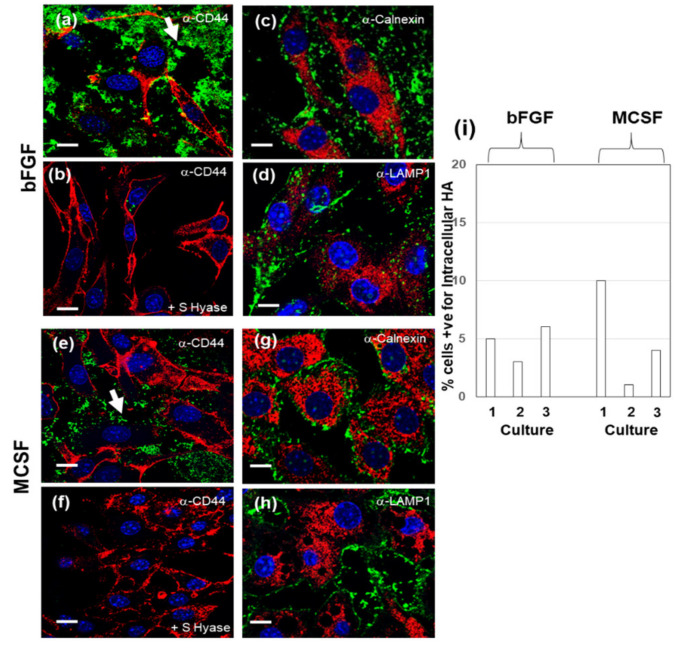
Effect of addition of exogenous HMW HA on association of endogenous HA with CD44, Calnexin and LAMP1 in LPS stimulated cultures. Cells treated with bFGF (**a**–**d**) or MCSF (**e**–**h**) were stimulated with LPS for 4 h, followed by 16 h incubation in basal FBS containing medium with 100 μg/mL HMW HA. The fixed cells were then dual labelled with bHABP and either anti-CD44 (**a**,**e**), anti-Calnexin (**c**,**g**) or anti-LAMP1 (**d**,**h**). Additional cells were pretreated with S Hyaluronidase prior to fixation, to remove extracellular prior to staining with bHAPB and anti-CD44 (**b**,**f**). The bar-graph in panel (**i**) shows the quantitation of cells positive for intracellular HA, which was determined as described in the Methods Section. Space bars = 10 μm. White arrowheads indicate the deposition of extracellular HA in between cell groups.

Cultures that were supplemented with HMW HA were also examined by confocal microscopy for HA and counterstained either with anti-CD44, anti-Calnexin or anti-LAMP1 (Figure 4 and Figure 5). In both non-stimulated (Figure 4) and LPS stimulated cultures (Figure 5), HA was deposited in between cells, on the growth surface (Figure 4 and Figure 5, panels a (bFGF), panels e (MCSF). *S Hyase* pretreatment of the cells (Figure 4 and Figure 5, panels b for bFGF, panels f for MCSF) completely removed any HA staining, including intracellular HA, showing that the addition of HMW HA to these cultures resulted in the depletion of the intracellular pool. Counterstaining for Calnexin (Figure 4 or Figure 5 panels c and g, bFGF and MCSF, respectively) or for LAMP1 (Figure 4 or Figure 5 panels d and h, bFGF and MCSF respectively) confirmed the depletion of intracellular HA. Furthermore, none of the treatments resulted in any detectable changes in the staining intensity or pattern of the two intracellular organelle associated proteins.

### 3.4. Effect of Addition of Exogenous HMW HA on TLR and Phagocytosis Pathway Genes in Basal and LPS Stimulated Cultures

**Figure 6:**
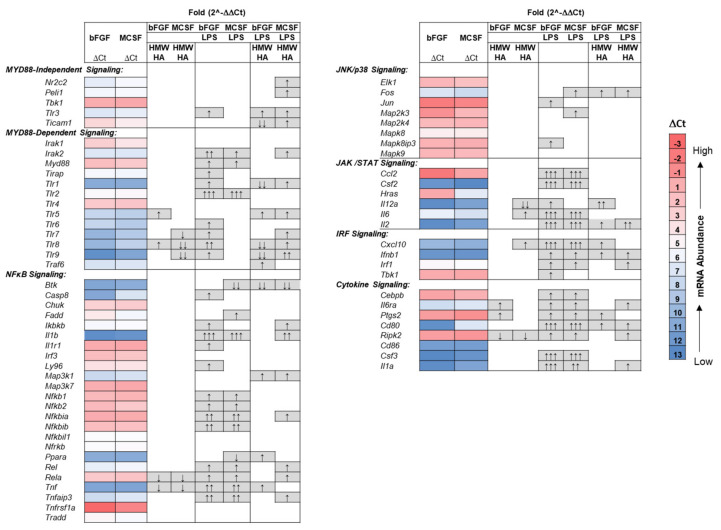
Heatmap of gene expression in the TLR signaling pathway in FLSC cultures maintained in bFGF or MCSF and their modulation by LPS, exogenous HMW HA, or a combination thereof. Cell layers from three independent culture preparations were assayed as described in the Methods. Fold changes relative to bFGF or MSCF only treated cultures were calculated from the respective ΔΔCt values as 2^−ΔCt^ and evaluated for statistical significance as described in the Methods. *B2m* was used as housekeeping gene. ↑ or ↓ = 2–5-fold increase or decrease; ↑↑ or ↓↓ = 5–50-fold increase or decrease; ↑↑↑ or ↓↓↓ > 50 fold increase.

**Figure 7:**
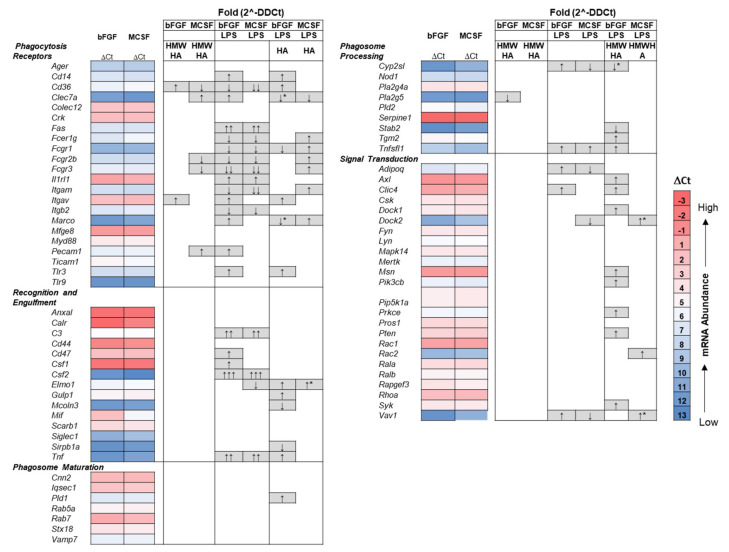
Heatmap of gene expression in the phagocytosis pathway in FLSC cultures maintained in bFGF or MCSF and modulation of their expression by LPS, Exogenous HA or a combination thereof. Cell layers from three independent cultures (as for Figure 6) were assayed as described in the Methods. Fold changes relative to bFGF or MSCF only treated cultures were calculated from the respective ΔΔCt values as 2^−ΔΔCt^ and evaluated for statistical significance as described in Section 2. *B2m* was used as housekeeping gene. ↑ or ↓ = 2–10-fold increase or decrease; ↑↑ or ↓↓ = 11–50-fold increase or decrease; ↑↑↑ or↓↓↓ > 50 fold increase. * Marks genes that were changed by exogenous HMW HA from LPS modulated levels to baseline levels (bFGF or MCSF).

These profound effects of exogenous HMW HA on expression levels of several hyaluronidases (Table 2 and Table 3) and on the depletion of the intracellular HA pool could result from modulation of the TLR4 signaling pathways [45,46,47] and phagocytic/degradative pathways [48,49] in the FLSC cultures. We examined the effect of HMW HA on multiple genes in these two pathways using QPCR array assays (see Table S1). The data are summarized as heatmaps in Figure 6 and Figure 7. For the TLR signaling pathway, there were no major differences in baseline expression between bFGF and MCSF treated cells (ΔCt, heatmap illustration, Figure 6), except for three genes, *Casp8* (Caspase 8), *Hras*, (HRas Proto-Oncogene, GTPase), and *Cd80* (in NFκb, JAK/Stat, and cytokine signaling, respectively), which were 3–4 fold different between the two growth factor treatments. After adding HMW HA to basal media, some minor effects on expression levels were seen for *Tlr-5*, *-7*, *-8*, *-9* (Toll Like Receptors 5,7,8 and 9), *Rela* (RELA Proto-Oncogene, NFκb Subunit) *Tnf*, *Il12*(Interleukin 12), *Il6*, *Il6ra* (Interleukin 6 Receptor Antagonist), *Ptgs2* (Prostaglandin-Endoperoxide Synthase 2), and *Ripk2* (Receptor Interacting Serine/Threonine Kinase 2), with both increases and decreases were seen for this group of genes.

As expected, the addition of LPS resulted in a strong modulation of a wide range of genes and in all TLR-dependent signaling pathways, for both bFGF and MCSF maintained cells. The most pronounced effects (>50 fold changes) were for *Tlr2*, *Il6*, *Il2*, *Ccl2* (C-C Motif Chemokine Ligand 2)*, Csf2* (Colony Stimulating Factor 2), *Cd80*, *Csf3* (Colony Stimulating Factor 3), and *IL1a*, all known to be modulated in multiple cells types after TLR4 activation by LPS. Notably, genes in the MYD88-independant or JNK/p38 signaling pathways were only minimally affected.

The addition of HMW HA to the LPS treated cultures resulted in additional modulation of many of the endotoxin affected genes. These were throughout all signaling pathways, including the MYD88-independent pathway. Furthermore, the majority of HMW HA-affected genes showed an increase in mRNA abundance above the levels already stimulated by LPS, except for *Tcam1* (Testicular Cell Adhesion Molecule 1), *Tlr1* (Toll-Like Receptor 1), and *Btk* (Bruton Tyrosine Kinase), which were strongly suppressed by addition of HMW HA.

As for the TLR signaling pathway genes, there were no significant differences between the baseline-expression of the phagocytic pathways genes, except for expression of *Mif* (Macrophage Inhibitory Factor), which was significantly lower in MCSF treated cells (∆Ct, heatmap illustration, Figure 7). HMW HA addition to the unstimulated cells resulted in only minor responses (~2 fold increase or decrease) for genes encoding phagocytosis receptors. The stimulation of cells with LPS broadly modulated these receptor genes, with most pronounced effects on *Fas* (Fas Cell Surface Death Receptor) (>10 fold increase) and *Fcgr3* (Fc Fragment of IgG Receptor) (>100 fold decrease). LPS also changed the expression of genes in the recognition and internalization pathway, with >10 fold activation of *C3* (Complement C3) and *Tnf* as well as >100 fold activation of *Csf2*. Other downstream genes for intracellular pathways were only mildly affected.

The addition of HMW HA also modulated many of the LPS-affected genes, with such effects seen throughout all functional gene groupings and also included 13 genes in the phagosomal process (*Cd14* (CD14 Molecule), *Itgav* (Integrin Subunit Alpha V), *Tlr3* (Toll-like Receptor 3), *Gulp1* (GULP PTB Domain Containing Engulfment Adaptor 1), *Mcoln3* (Mucolipin 3), *Sirpb1a* (Signal Regulatory Protein Alpha), *Pld1* (Phospholipase D1), *Stab2* (Stabilin 2), *Tgm2* (Transglutaminase 2), *Axl* (AXL Receptor Tyrosine Kinase), as well as five genes in the downstream signaling that were not changed by LPS alone, (*Msn* (Moesin), *Pik3cb* Phosphatidylinositol-4,5-Bisphosphate 3-Kinase Catalytic Subunit Beta)*, Prkce* (Protein Kinase C Epsilon, *Pten* (Phosphatase and Tensin Homolog), and *Syk* (Tyrosine Kinase). Furthermore, HMW HA restored several phagocytosis genes (*Clec7a* (C-Type Lectin Domain Containing 7A), *Marco* (Macrophage Receptor With Collagenous Structure), *Elmo1* (Engulfment And Cell Motility 1), and *Doc2* (Double C2 Domain Alpha)) modulated by LPS back to baseline levels of bFGF or MCSF treated cultures (* marked in Figure 7).

### 3.5. Effect of LPS and Exogenous HMW HA on VCAN, ACAN, and PRG4 Gene Expression and Protein Levels

We next investigated whether exogenous HA modulates the expression, production, and secretion of other macromolecules. We focused on VCAN and ACAN, two HA-binding proteoglycans known to be products of fibroblastic cells [35,50,51], as well as PRG4, which is also a well characterized synovial fluid component synthesized by synovial cells [37,52]. The expression levels for all three macromolecules were robust, with ∆Ct values of ~6–9 for *Acan*, ~4–6 for *Vcan*, and ~3–4 for *Prg4*. No significant differences were seen between bFGF and MCSF treated cells, and the expression levels were similar when assayed after 4 or 16 h post-medium change (Table 4).

The addition of LPS (Table 5) had a pronounced effect on mRNA levels of *Acan*, with ~4–6 fold decreases in the presence of either growth factor. These decreases remained over the 16 h post LPS stimulation and agrees with the well-documented depression of ACAN gene expression and synthesis in chondrogenic cells. In contrast, VCAN gene expression was not affected during the acute LPS treatment, but then increased ~2 fold during the subsequent 16 h incubation for both growth factor conditions. *Prg4* expression was not significantly altered during or post-LPS treatment in either bFGF or MCSF supplemented cultures.

Addition of exogenous HMW HA (Table 6) resulted in a rather variable response on *Acan* and *Vcan* expression levels, with an upward trend in non-LPS treated cultures for both genes. By comparison, in LPS stimulated cultures, only *Acan* levels trended upwards (~1.6–2 fold), whereas *Vcan* levels trended downwards (~2 fold), although those effects were not statistically significant. *Prg4* expression was not affected by HMW HA addition in any of the culture conditions.

**Figure 8:**
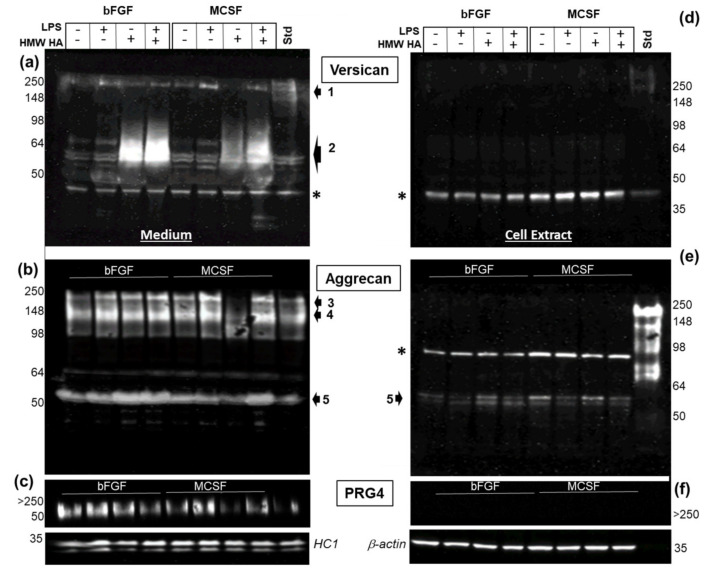
Western analysis of VCAN, ACAN, and PRG4 from FLSC cultures maintained in bFGF or MCSF, stimulated or not with LPS and exposed or not to exogenous HMW HA. Conditioned media (panels **a**–**c**) and cell extracts (panels **d**–**f**) were collected and prepared for SDS PAGE/Western blotting as described in the Methods. Membranes were probed for VCAN with anti-DPE (**a**,**d**), and for with a mix of anti-CDAG and anti-DLS (1 μg/mL each) for ACAN (**b**,**e**). The later were reprobed for PRG4 (panels **c**,**f**) with MAb 9G3 as described in the Methods. To confirm equivalent loading between samples, all membranes from media were finally with anti-HC1 and cell extract samples with anti-β-actin (bottom panels). Identified VCAN species 1and 2 represent the high molecular weight core protein and the ADAMTS-generated G1 fragment, respectively. Identified ACAN species include the full length and C-terminally processed core protein, respectively, and the ADAMTS-generated G1 fragment (5). Non-specific reactive bands are indicated by **a** (*).

We performed Western blot analyses of cell extracts and media for all three macromolecules to examine the effect of the various culture conditions on core protein expression (Figure 8). For each protein, products were predominantly found in the medium compartment (Figure 8a–c). For ACAN and VCAN, immunoreactive species were consistent with both full length and the ADAMTS-generated G1 products, and only the intact form of PRG4 was detected. Most notable was the effect of HMW HA on the secretion of the DPE-reactive VCAN-G1 into the medium (Figure 8a), with no detectable effects on either CDAG/DLS reactive ACAN or Mab 9G3-reactive PRG4 (Figure 8 panels b and c, respectively). A difference in VCAN products due to variable loading artifacts could be ruled out, because the reactivity of the FBS-derived HC1 fragment was constant across wells.

**Figure 9:**
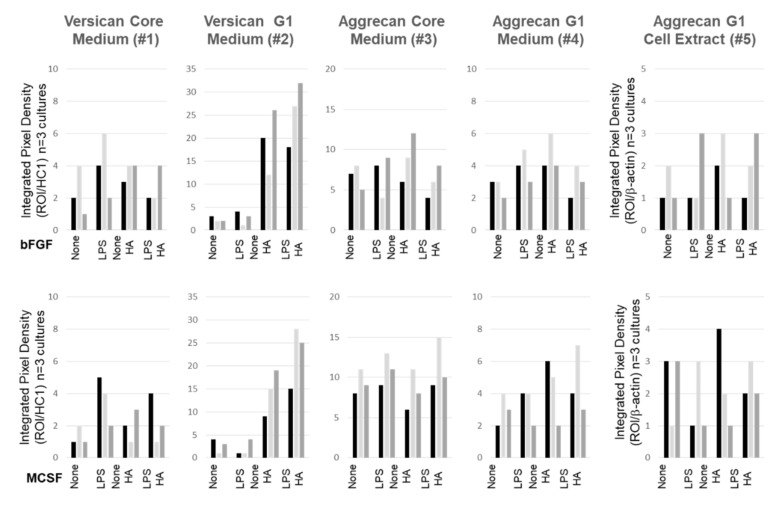
Densitometry Quantitation of Immunoreactive VCAN and ACAN species present in medium and cell layer compartments of three separately prepared FLSC cultures treated as described for Figure 8. Each cell preparation is indicated by differently shaded bars. Data were collected using Image J software and are expressed as Integrated Pixel Density of immunoreactive bands relative to Integrated Pixel density of HC1 reactive bands for medium samples or of b-actin reactive bands for cell extract samples.

For cell layer extracts, only the CDAG/DLS- A disintegrin and metalloproteinase with thrombospondin motifs (ADAMTS)-generated G1 fragment of ACAN was detected, and this product was somewhat decreased in LPS supplemented cultures. No effect of exogenously added HMW HA was detectable. VCAN or PRG4 in cell extracts were below the detection limits of the Western assay. Furthermore, the gel loading levels (per cell density) were equivalent for all samples, as shown by the constant reactivity of β-actin in all of the samples. The data shown are typical of three separate cell preparations, and integrated pixel density measurement of immunoreactive ACAN and VCAN species was performed on the additional western blot images using Image J. These data are shown in Figure 9, and clearly support our findings that the addition of high HMW HA resulted in a dramatic increase in the secretion of the ADAMTS-generated G1-product of VCAN V0/V1.

### 3.6. Confocal Localization of VCAN and ACAN before and after Addition of Exogenous HMW HA

**Figure 10:**
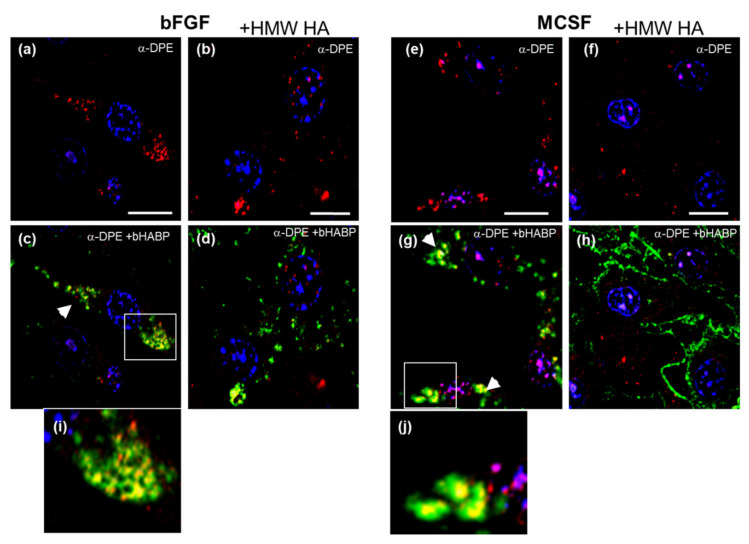
Localization of VCAN, bFGF, and MCSF treated FLSC cultures before and after addition of exogenous HMW HA. Cells were dual labelled with anti-DPE (red fluorescence, panels **a**,**b**,**e**,**f**) and bHABP (green fluorescence panels **c**,**d**,**g**,**h**). Intracellular HA co-localized with VCAN is marked in panels **c**,**d**, and g with white arrow heads (yellow fluorescence). ~50% of cells in a given imaged area was positive for VCAN, but the degree of staining varied between cells, as illustrated in panels **a**,**b**,**e**,**f**. Higher magnification images of co-localized areas in panels (**c**,**g**) are shown in panels (**i**,**j**), respectively.

We performed dual staining with bHABP and anti-DPE (Figure 10) or anti-CDAG/DLS to examine the possibility of a molecular association between endogenous HA and VCAN or ACAN (Figure 10). Notably, immuno-reactive VCAN was distributed in the cell body in both bFGF and MCSF treated cultures (Figure 10, panels a and e, respectively), and this was clearly co-localized with the cell-associated HA (Figure 10, panels c,i and g,j respectively). The addition of HMW HA resulted in a significant reduction of the cell-associated VCAN staining (Figure 10b,f) and a concomitant loss (Figure 10d,h) of its co-localization with HA. This is consistent with a HMW HA-induced secretion of cell-associated DPE-reactive VCAN-G1 into the culture medium during the 16 h treatment period (Figure 8a).

**Figure 11:**
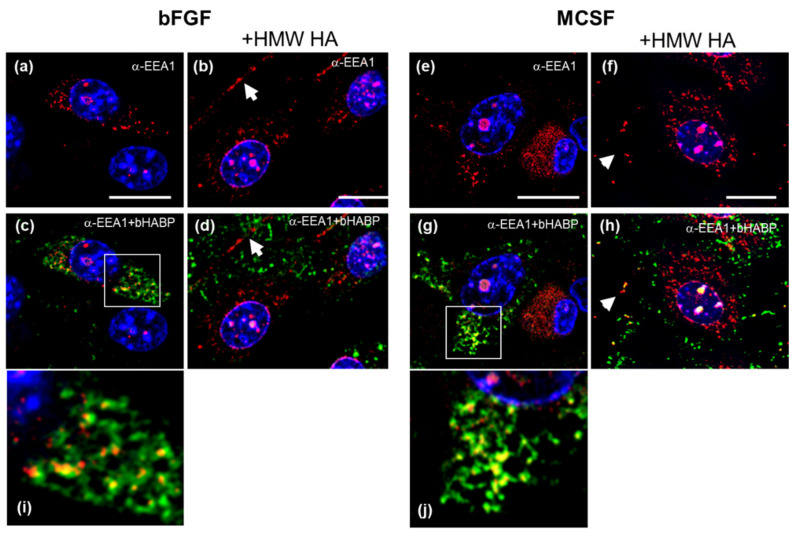
Co-localization of Early Endosomal Marker (EEA1) and HA in bFGF and MCSF treated FLSC cultures before and after addition of exogenous HMW HA. Cells were dual labelled with bHABP (green fluorescence) and anti-EEA1 (red fluorescence). Intracellular HA localized with early endosomes is marked in panels c and g (yellow fluorescence), ~50% of cells in a given imaged area was positive for VCAN, but the degree of staining varied between cells, as illustrated in panels **a**,**b**,**e**,**f**. Higher magnification images of colocalized areas in panels (**c**,**g**) are shown in panels (**i**,**j**), respectively. Some EEA1 reactivity appeared to be localized away from the cell body, after exposure to exogenous HMW HA (marked with a white arrowhead in panels **d**,**h**).

To assess whether the mechanism for VCAN and intracellular HA export might involve endosomal/EV secretory pathways [53], we immunostained cells for early endosome antigen (EEA) (Figure 11). A portion of the intracellular HA was co-localized with EEA- positive vesicles (Figure 11c,g,i,j, bFGF and MCSF treated, respectively). The intracellular co-localization was abolished after exposure to HMW HA, and both HA and as well as EEA1 protein were now in the extracellular space (white *, panels d and h). Notably the intracellular vesicular staining pattern for EEA (Figure 11a,b,e,f) was also seen for the VCAN-DPE epitope (Figure 10a,b,e,f), suggesting that it is in the same endosomal compartment as the EEA1 protein.

**Figure 12:**
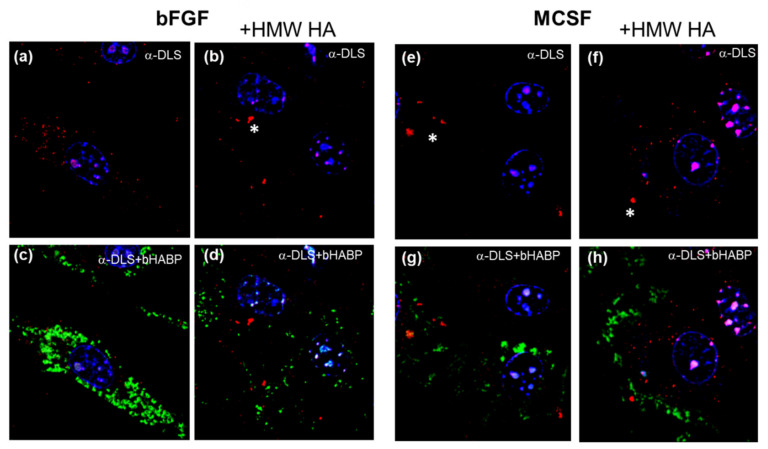
Localization of ACAN and HA in bFGF and MCSF treated FLSC cultures before and after treatment with exogenous HMW HA. The cells were dual labelled with anti-DLS (red fluorescence, panels **a**–**h**).and bHABP (green fluorescence panels **c**,**d**,**g**,**h**). ~20% of cells in a given imaged area stained positive for ACAN and no co-localization between HA and ACAN was observed.

ACAN immunoreactive species were also found in association with cells in bFGF or MCSF treated cultures (Figure 12a,e) and, in some cells, these were what appeared to be multi-molecular aggregates (white * in Figure 12). However, no co-localization with HA was detected, nor did the addition of HMW HA result in an increased shedding of cell-associated ACAN. This was consistent with the Western analyses that are shown in Figure 8b.

## 4. Discussion

Because much of the published work on molecular mechanisms of action of IA HMW HA does not describe its effects on endogenous HA metabolism in joint tissues, we used a model cell system, to examine the effect of HMW HA on endogenously synthesized HA by FLSCs derived from adult murine IFP. The choice of cells was based on our previous reports that the injection of HMW HA into an inflamed mouse knee joint is contained predominantly to the femora-patellar compartment where it affects the inflammatory and downstream fibrotic response of the synovial lining and underlying adipose tissue [21,54]. The IFP is localized in that joint compartment and it is readily dissected from the mouse to provide a reproducible source for cell isolation and subsequent culture. The IFP, although generally considered an ‘extra synovial tissue’, has recently attracted attention with regards to the role of inflammation and pain in human OA development and progression (reviewed in [55]). Firstly, it contains a cell population at its surface that resembles those lining the synovium, being responsible for the production of HA and PRG4 in joint fluid. Secondly, the autocrine properties of adipose tissue with its resident multipotent stromal cells can serve as a source of inflammatory mediators, such as interleukins, nitric oxide, chemokines, and prostaglandins [56]. Thirdly, this multipotent cell population has been described in post-injury responses, including cellular proliferation, HA accumulation, and fibrotic remodeling, as well as having a potential for neo-vascularization [57], chondrogenesis [58], or modulation of pain responses [59]. Notably, such multipotent properties have also been reported for cells in synovial membranes [60,61,62] and the superficial zone of articular cartilage [63].

The murine FLSC cultures used in this study display many characteristics of multipotent progenitor cells isolated from other adipose tissue stroma or bone marrow. For example, these cells rapidly proliferate in bFGF supplemented medium [64] and they express stem cells markers, such as Sca1 and CD44, as well as adipogenic, fibrogenic, and chondrogenic genes. Morphologically, they resemble cultures that are described as “Fibroblast-Like Synoviocytes”, which are from synovium [65] and they also produce PRG4, [66,67]. Cell shape differences were observed between bFGF and MCSF (a known inducer of M1 macrophage activation [68,69]), as well as in the expression of mesenchymal markers (*Col1a1* and *Itgam*) and macrophage markers (*Emr1* and *Cd163*). However, the same robust response to LPS was seen with either growth factor. The endotoxin activated multiple proinflammatory genes in the TLR-pathway as well as genes for phagocytosis receptors and engulfment. Several of these (*Ccl2*, *Tnf*, and *IL 1b*) have also been reported to be elevated in IFP tissue from a murine model of OA [69]. This further supports the relevance of using this culture system to provide new information of the potential mechanism of exogenous HMW HA on cell populations in a synovial joint.

HA production by synovium derived cell cultures from either OA or RA patients have been extensively studied in connection with the production of inflammatory molecules and therapeutic interventions with a variety of small molecule drugs [70,71,72] to control inflammation in a range of arthritic conditions. Furthermore, such cells have been used to examine the effect of potential therapeutic HA formulations [73]. With the FLSC cultures, we also observed a robust effect of HA on mRNA levels for *IL6* (increased ~2 or 20–40 fold in unstimulated or LPS activated cells, respectively) and LPS-induced *Nos2* (decreased ~33 fold or ~7 fold for bFGF or MCSF, respectively). To our knowledge no previous reports have described the impact of exogenous HMW HA on endogenous HA metabolism. Thus, when we examined for effects on the expression of genes for HA synthesis, ECM assembly, and HA degradation, only cells that were activated with LPS responded. This was seen as an increase in mRNA levels for *Has2*, and three hyaluronidases, *Hyal1*, *Hyal2*, and *Tmem2*, suggesting an increase in turnover (both synthesis and degradation) of HA. The amount of HA synthesized in these cultures was not determined; however, it should be noted that in addition to changes in mRNA abundance of the synthases, HA production will be likely a combination of transcriptional as well post-translational modification of HAS enzymes [74]. Our results are supported by reports of increased catabolism by hyaluronidases with tissue inflammation [75,76]. In addition to the change in mRNA levels for the hyaluronidases, endogenously synthesized HA was localized to intracellularly to endosomal/lysosomal compartments. Most significantly, the addition of exogenous HA eliminated these intracellular pools in both the absence and presence of LPS stimulation. Whether the accumulation of intracellular HA is due to recycling of newly synthesized HA via phago/endocytosis [77] involving CD44 [78] and/or Stabilin 2 [79] remains to be established. Thus, alternatively, these vesicular structures could be the result of fusion of auto-phagosomes with lysosomes [80]. However, the induction of autophagy and intracellular HA accumulation were seen only under hyperglycemic (25 mM glucose) culture conditions [40], whereas physiological concentrations of glucose (5 mM) were used throughout all of the experiments reported here.

This study also showed an increased secretion of the DPE-VCAN-G1 fragment into the culture medium of cells that were maintained in the presence of exogenous HA. Moreover, the data showing intracellular VCAN species co-localized with HA in early endosomal compartments support the notion that both macromolecules are released into the medium by the same mechanism, and accelerated by exogenous HMW HA. Early endosome fusion with the plasma membrane could result in such an alternate export/secretion pathway for both HA and VCAN products, as has been reported for cell surface receptors [81]. It should be noted that VCAN is a regulatory ECM component for inflammation responses by mesenchymal cells, such as vascular [82] or gut [83] derived smooth muscle cells, as well as macrophages [84] and T-lymphocytes [85]. However, the current experimental system does not provide information on the levels of secretion of endogenous HA (ng/mL range [35]) into the culture medium due to the high concentration (100 μg/mL) of exogenously added HMW HA. It should also be noted that IA-HA injections that are available for the treatment of OA vary greatly in structure and it is not known whether such differences may produce different outcomes.

Confocal localization studies only detected a minor association of endogenously produced HA with CD44 at the cell surface, and this was not altered after addition of HMW HA. Moreover we did not observe TLR2 or TLR4 protein co-localized with endogenous or exogenous HMW HA, supporting the absence of a physical association between either HA and these receptors to mediate downstream signaling. However, after HMW HA addition, we observed increased HA deposition in between the cells, in a ‘carpet-like-fashion’. A possible explanation for a mechanism of action of exogenous HMW HA on these FLSC might therefore be through bulk physicochemical [86], rheological [87], and osmotic properties [88]. Indeed, inflammatory signaling through CD44, TLRs or other cytokine receptors [89,90,91], as well as endosomal recycling and micro vesicular secretion are regulated by pH [92] and Ca^2+^ flux [93,94,95].

## 5. Conclusions

In the in vitro inflammation model system, exogenous HMW HA was not associated with cell surface CD44, TLR2, or TLR4 and, thus, the effects on gene modulation are unlikely to be mediated through these receptors. It should be noted that our current study did not examine and HA receptors, such as Layilin [96] and RHAMM [97] which have been implicated in cell migration and fibrosis, were not examined. Additional investigations should be conducted in order to assess the effect of exogenous HMW HA in cell culture model systems of migration and fibrosis responses.

Because the most pronounced effect of exogenous HMW HA appeared to be on vesicular pathways, a better understanding of the involvement of altered micro-vesicular trafficking [95] and secretion by mesenchymal progenitor cells [98] also abundant in joint tissues [99] may provide critical information for optimizing the actions of therapeutic HA preparations for clinical use disease-modifying agents for degenerative joint disease.

## Figures and Tables

**Table 1:** Baseline Expression levels of TLR4 responsive genes, *Il6* and *Nos2* and genes involved in HA synthesis, extracellular organization and degradation at 4 and 16 h post-medium change.

Gene	4 h + bFGF	4 h + MCSF	36 h + bFGF	36 h + MCSF
	⚜Ct *	⚜Ct *	⚜Ct *	⚜Ct *
*Il6*	10.88 (± 2.17)	11.28 (± 2.54)	12.78 (±1.55)	13.15 (± 2.00)
*Nos2*	ND	ND	ND	ND
*Has 1*	7.64 (± 1.01)	8.11 (± 1.92)	8.83 (±0.15)	9.22 (± 0.93)
*Has2*	8.33 (± 1.48)	8.09 (± 2.31)	8.52 (±0.92)	7.65 (± 1.32)
*Ptx3*	8.19 (± 2.56)	6.07 (± 3.21)	8.35 (±0.68)	8.92 (± 1.29)
*Tnfaip6*	9.96 (± 2.01)	9.28 (± 1.89)	12.68 (±1.49)	13.13 (± 2.19)
*Cd44*	1.14 (± 0.31)	1.67 (± 0.52)	1.26 (±0.41)	1.41 (± 0.31)
*Hyla1*	9.05 (± 2.11)	8.52 (± 2.42)	7.33 (±0.46)	7.12 (± 0.68)
*Hyal2*	5.39 (± 1.11)	4.94 (± 0.76)	4.34 (±0.28)	3.97 (± 0.63)
*Cemip*	8.81 (± 2.25)	6.82 (± 1.13)	7.53 (±0.52)	7.82 (± 0.67)
*Tmem2*	6.26 (± 0.99)	6.05 (± 1.26)	9.84 (±0.69)	8.17 (± 0.88)

* Data shown are the mean (± SD) from triplicate cultures of 2 separately prepared cultures. *B2m* was used as housekeeping gene. ND = Not detected (Ct > 14.0).

**Table 2:** Fold changes in Expression of TLR4 responsive genes *Il6*, *Nos2*, and genes involved in HA synthesis, extracellular organization and degradation following a 4 h LPS stimulus or a 4 h LPS Stimulus followed by a 16 h incubation in complete medium.

	4 h bFGF + LPS	4 h MCSF + LPS	+16 h bFGF	+16 h MCSF
Gene	* FOLD	FOLD	FOLD	FOLD
*Il6*	101.5 (± 0.92)	93.4 (± 3.44)	155.1 (± 22.13)	76.64 (± 11.11)
*Nos2*	** >>100	>>100	>>100	>>100
*Has1*	1.98 (± 0.21)	2.90 (± 0.87)	4.85 (± 0.58)	8.90 (± 0.13)
*Has2*	1.62 (± 0.03)	1.65 (± 0.11)	0.69 (± 0.11)	0.36 (± 0.08)
*Ptx3*	12.40 (± 2.33)	5.32 (± 0.82)	66.67 (± 4.88)	132.5 (± 25.3)
*Tnfaip6*	6.09 (± 1.20)	12.62 (± 2.11)	4.97 (± 0.81)	7.89 (± 0.39)
*Cd44*	1.04 (± 0.32)	1.49 (± 0.46)	0.95 (± 0.28)	1.23 (± 0.41)
*Hyla1*	0.86 (± 0.14)	0.59 (± 0.32)	0.45 (± 0.12)	0.28 (± 0.03)
*Hyal2*	0.85 (± 0.29)	0.43 (± 0.08)	0.28 (± 0.11)	0.11 (± 0.06)
*Cemip*	1.57 (± 0.22)	0.88 (± 0.71)	1.30 (± 0.21)	0.78 (± 0.39)
*Tmem2*	1.50 (± 0.41)	1.14 (± 0.15)	0.24 (± 0.08)	0.30 (± 0.06)

* Fold change values were calculated from the ∆∆Ct values of No LPS vs. +LPS stimulus as 2^−∆∆Ct^. The mean (±SD) from triplicate cultures of two separately prepared cultures. *B2m* was used as housekeeping gene. ** ∆Ct values increased from >14.0 to ~4.0.

**Table 3:** Fold changes in the expression of TLR4 responsive genes, *Il6*, *Nos2* genes involved in HA synthesis, extracellular organization and degradation following a 4 h incubation in basal or LPS supplemented medium, followed by a 16 h incubation in complete medium containing 100 μg/mL HMW HA.

	4 h Basal Medium		4 h LPS Treatment	
	16 h bFGF + HA	16 h MCSF + HA	16 h bFGF + HA	16 h MCSF + HA
Gene	* FOLD	FOLD	FOLD	FOLD
*Il6*	^#^ 2.58 (± 1.07)	^#^ 2.29 (± 0.84)	^###^ 21.4 (± 4.5)	^###^ 44.3 (± 5.99)
*Nos2*	ND	ND	^###^ 0.03 (± 0.06)	^###^ 0.14 (± 0.03)
*Has1*	0.86 (± 0.41)	1.38 (± 0.51)	1.06 (± 0.21)	1.04 (± 0.23)
*Has2*	1.08 (± 0.33)	1.44 (± 0.59)	^#^ 1.97 (± 0.31)	^#^ 1.87 (± 0.29)
*Ptx3*	0.87 (± 0.64)	1.01 (± 0.14)	1.08 (± 0.09)	1.13 (± 0.25)
*Tnfaip6*	0.58 (± 0.44)	1.10 (± 0.61)	1.33 (± 0.26)	1.25(± 0.44)
*Cd44*	1.23 (± 0.29)	1.14 (± 0.17)	0.97 (± 0.31)	1.19 (± 0.29)
*Hyal1*	1.12 (± 0.44)	1.13 (± 0.22)	^#^ 2.01 (± 0.14)	^##^ 2.68(± 0.27)
*Hyal2*	0.60 (± 0.71)	1.27 (± 0.41)	^#^ 1.95 (± 0.54)	^##^ 3.29 (± 0.98)
*Cemip*	1.09 (± 0.32)	0.97 (± 0.31)	0.96 (± 0.33)	1.15 (± 0.66)
*Tmem2*	0.68 (± 0.58)	0.74 (± 0.29)	^#^ 1.78 (± 0.11)	^##^ 2.32 (± 0.28)

Fold values were calculated from the ∆∆Ct values of +HA vs. −HA supplemented cultures. The mean (±SD) from triplicate cultures of 2 separately prepared cultures. *B2m* was used as housekeeping gene. ND = Not detected. ^#^ *p* < 0.05; ^##^ *p* < 0.01; ^###^ *p* < 0.001.

**Table 4:** Baseline expression of Aggrecan (*Acan*), versican (*Vcan*), and *Prg4* at 4 and 16 h post-medium change.

Gene	4 h + bFGF	4 h + MCSF	36 h + bFGF	36 h + MCSF
	⚜Ct *	⚜Ct *	⚜Ct *	⚜Ct *
*Acan*	9.12 (± 1.1)	6.93 (± 0.82)	8.97 (± 1.6)	6.42 (± 0.61)
*Vcan*	6.91 (± 0.48)	4.76 (± 1.01)	6.29 (± 0.57)	4.74 (± 2.01)
*Prg4*	3.55 (± 0.51)	3.92 (± 0.21)	3.67 (± 0.86)	3.19 (± 0.20)

* Data shown are the mean (±SD) from triplicate cultures of 2 separately prepared cultures. *B2m* was used as a housekeeping gene.

**Table 5:** Fold changes in Expression of *Acan*, *Vcan*, and *Prg4* after 4 h of LPS stimulation followed by 16 h incubation in complete medium.

Gene	4 h bFGF + LPS	4 h MCSF + LPS	+ 16 h bFGF	+ 16 h MCSF
	* FOLD	FOLD	FOLD	FOLD
*Acan*	^##^ 0.24 (± 0.11)	^##^ 0.29 (± 0.03)	^##^ 0.31 (± 0.08)	^##^ 0.09 (± 0.03)
*Vcan*	0.84 (± 0.37)	1.44 (± 0.38)	^#^ 2.44 (± 0.41)	1.03 (± 0.38)
*Prg4*	0.74 (± 0.18)	0.78 (± 0.34)	^#^ 2.07 (± 4.88)	0.94 (± 25.3)

* Fold values were calculated from the ∆∆Ct values of basal vs. +LPS stimulus as 2^−∆∆Ct^. The mean (±SD) from triplicate cultures of 2 separately prepared cultures. ^#^ *p* < 0.05; ^##^ *p* < 0.01.

**Table 6:** Fold changes in Expression of *Acan*, *Vcan*, and *Prg4* following a 4 h incubation in basal or LPS supplemented medium, followed by a 16 h incubation in complete medium containing 100 μg/mL HMW HA.

Gene	bFGF + HA	MCSF + HA	bFGF + LPS + HA	MCSF + LPS + HA
	* FOLD	FOLD	FOLD	FOLD
*Acan*	1.64 (± 0.22)	0.62 (± 0.62)	1.56 (± 0.51)	2.26 (± 0.61)
*Vcan*	1.76 (± 0.21)	2.59 (± 0.11)	0.78 (± 0.32)	0.40 (± 0.63)
*Prg4*	1.44 (± 0.25)	1.56 (± 0.31)	1.04 (± 0.08)	1.69 (± 0.21)

* Fold values were calculated from the ∆∆Ct values of +HA vs. −HA supplemented cultures as 2^−∆∆Ct^. *B2m* was used as the housekeeping gene. The mean (±SD) from triplicate cultures of 2 separately prepared cultures.

## Supplementary Materials

The following are available online at https://www.mdpi.com/2073-4409/9/7/1681/s1, Figure S1: Schematic of Culture Timeline and Treatment Methods. Figure S2: Expression of Multipotent Progenitor Cell Markers in IFP cultures maintained in bFGF or MCSF. Figure S3: Schematic of Epitope locations on Versican and Aggrecan Core Proteins for Peptide-Specific Antibodies used in western blots and confocal localization. The versican antibody anti-DPE recognizes a regions in the -GAG domain of V0/V1 isoform in both, the intact core protein and the ADAMTS-G1 cleavage product. The aggrecan antibody anti-CDAG recognize the globular G1 (HA binding) domain in the intact core protein and the ADAMTS-G1 cleavage product. The anti-DLS recognizes multiple epitope regions in the chondroitin sulfate (CS) domains 1 and 2. Figure S4: Western Blotting of HC1 in commercially available preparations of Fetal Bovine Serum used in this study. Ten μL of FBS were diluted with 90 μL 0.1 M ammonium acetate, pH 7.5. 50 μL portions were incubated at 37 °C for 20 min. in the absence (-) or presence (+) of Proteinase free Chondroitinase ABC, prior to SDS PAGE and western blotting with anti-HC antibody. Lanes 1, 2, 5, 6 show 2 different batches of FBS from Atlanta Biologics and lanes 3, 4, 7, 8 show 2 different batches of FBS from Sigma Aldrich. Table S1: Listing of single gene of Taqman Primers (a), Phagocytosis gene array (b) and TLR Signaling Gene Array. Table S2: Listing of Abbreviations used.
