# Supplementary material for: Addition of High Molecular Weight Hyaluronic Acid to Fibroblast-Like Stromal Cells Modulates Endogenous Hyaluronic Acid Metabolism and Enhances Proteolytic Processing and Secretion of Versican
Source: Cells. 2020 Jul 13;9(7):1681. doi: 10.3390/cells9071681 (PMC7407811; doi:10.3390/cells9071681)
Supplement: Supplementary file 1 [file cells-09-01681-s001.pdf]

cells

MDPI

Supplementary Materials:

The following are available online at www.mdpi.com/xxx/s1,

Figure S1: Schematic of Culture Timeline and Treatment Methods, **Figure S2**: Expression of Multipotent Progenitor Cell and Macrophage Markers in FLSC cultures maintained in bFGF or MCSF. **Figure S3:** Schematic of Epitope locations on Versican and Aggrecan Core Proteins for Peptide-Specific Antibodies used in western blots and confocal localization. **Figure S4:** Western Blotting of HC1 in commercially available preparations of Fetal Bovine Serum used in this study. **Table S1:** Listing of single gene Taqman Primers (a), Phagocytosis gene array (b) and TLR Signaling gene array. **Table S2**: Listing of Abbreviations used throughout the manuscript.

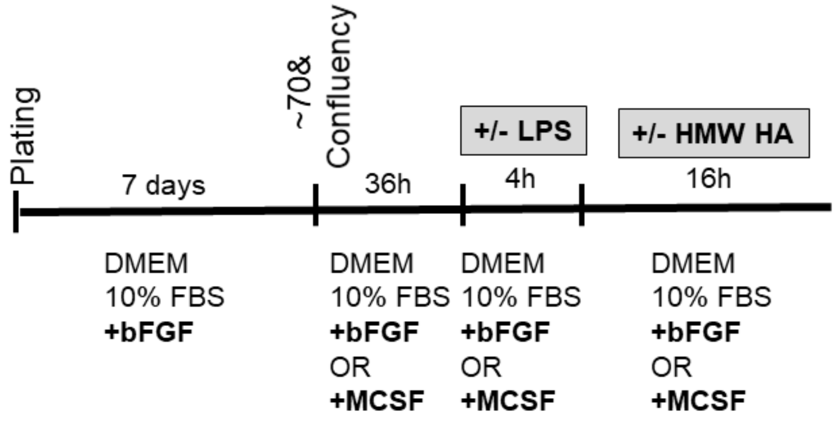

Figure S1. Schematic of Culture Timeline and Treatment Methods.

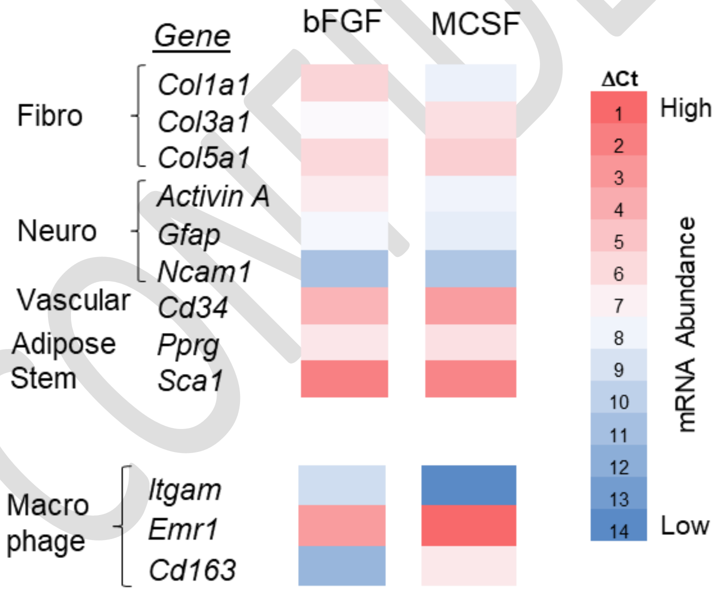

Figure S2. Expression of multipotent progenitor cell and macrophage markers in IFP cultures maintained in bFGF or MCSF.

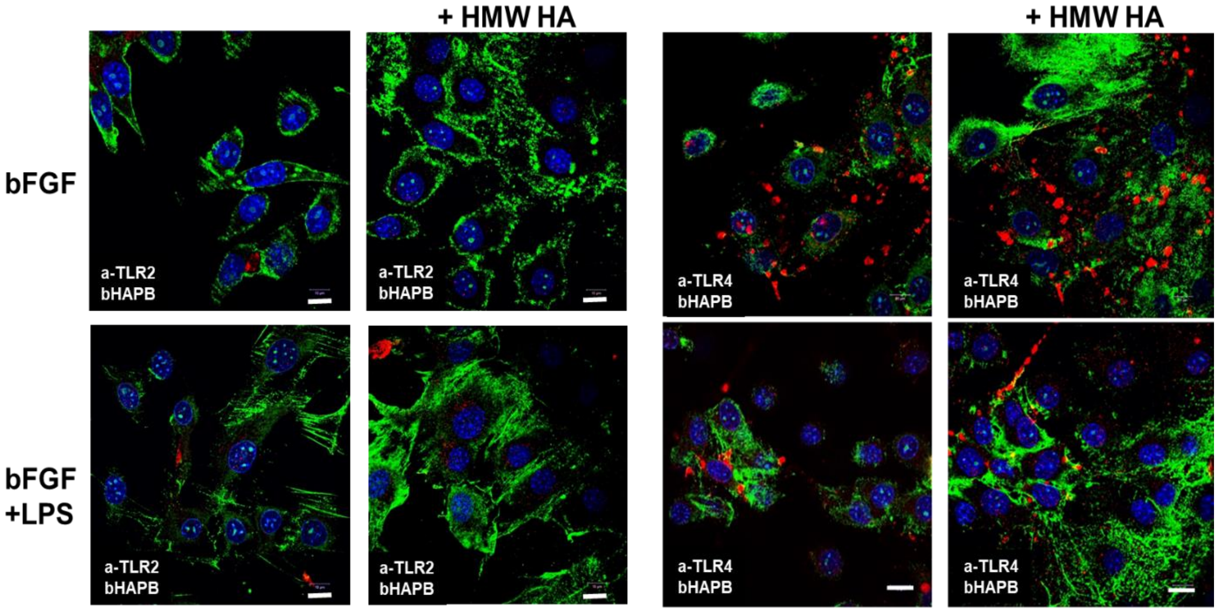

Figure S3. Confocal Localization of TLR2 or TLR4 Proteins (Red Fluorescence) and HA (green fluorescence) in FLSC Cultures treated or not with LPS followed by treatment or not with exogenous HMW HA. TLR2 protein was associated with very few cells (5-10% of cells +ve within an imaged filed), whereas TLR4 protein was more abundant, (40-50% of cells +ve within and imaged field). Neither protein abundances nor distributions were altered by addition of LPS and/or HMW HA. .

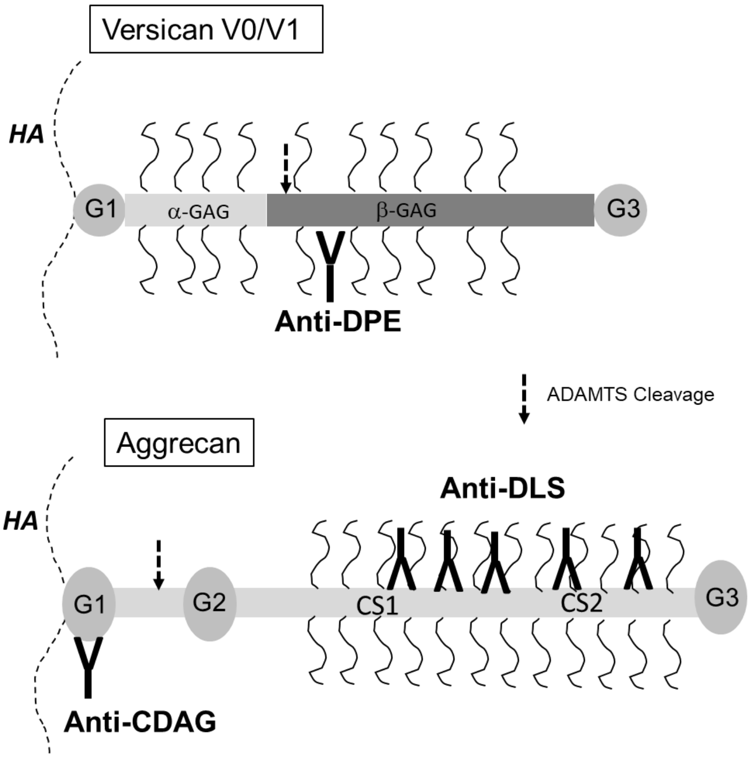

Figure S4. Schematic of Epitope locations on Versican and Aggrecan Core Proteins for Peptide-Specific Antibodies used in western blots and confocal localization. The versican antibody anti-DPE recognizes a regions in the β-GAG domain of V0/V1 isoform in both, the intact core protein and the ADAMTS-G1 cleavage product. The aggrecan antibody anti-CDAG recognize the globular G1 (HA binding) domain in the intact core protein and the ADAMTS-G1 cleavage product. The anti-DLS recognizes multiple epitope regions in the chondroitin sulfate (CS) domains 1 and 2.

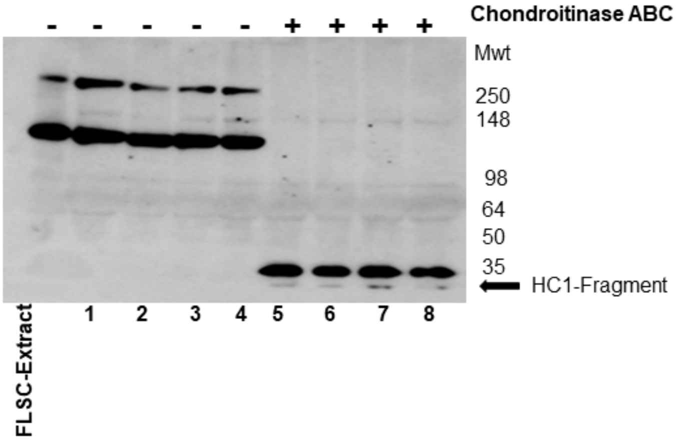

Figure S4. Western Blotting of HC1 in commercially available preparations of Fetal Bovine Serum used in this study. Ten uL of FBS were diluted with 90 uL 0.1 M ammonium acetate, pH 7.5. 50uL portions were incubated at 37 C for 20 min in the absence (-) or presence (+) of Proteinase free Chondrotinase ABC, prior to SDS PAGE and western blotting with anti-HC antibody. Lanes 1, 2, 5,6 show 2 different batches of FBS from Atlanta Biologics and lanes 3,4,7,8 show 2 different batches of FBS from Sigma Aldrich.

Table S1. Listing of single gene Taqman Primers (**a**), TLR Signaling gene array (**b**) and Phagocytosis gene array.

Protein:	Gene:	Taqman Primer:
Activin A	*Acvr1*	Mm01331069_m1
Aggrecan	*Acan*	Mm00545794_m1
CD163 Molecule	*Cd163*	Mm00474091_m1
CD34 Molecule	*Cd34*	Mm00519283_m1
CD44 Molecule	*Cd44*	Mm01277161_m1
Cell Migration Inducing Hyaluronidase	*Cemip*	Mm00472921_m1
Cell Surface Hyaluronidase	*Tmem2*	Mm00459599_m1
Collagen Type 1 alpha 1	*Col1a1*	Mm00801666_g1
Collagen Type 3 alpha 1	*Col3a1*	Mm00802300_m1
Collagen Type 5 alpha 1	*Col5a1*	Mm00489299_m1
EGF module-containing mucin-like receptor	*Emr1*	Rn01527631_m1
Glial Fibrillary Acidic Protein	*Gfap*	Mm01253033_m1
Hyaluronan Synthase 1	*Has1*	Mm03048195_m1
Hyaluronan Synthase 2	*Has2*	Mm00515089_m1
Hyaluronan Synthase 3	*Has3*	Mm00515092_m1
Hyaluronidase 1	*Hyal1*	Mm00476206_m1
Hyaluronidase 2	*Hyal2*	Mm01230688_g1
Integrin Subunit Alpha M	*Itgam*	Mm00434455_m1
Interleukin 6	*Il6*	Mm00446190_m1
Lubricin	*Prg4*	Mm01284582_m1
Neural Cell Adhesion Molecule 1	*Ncam1*	Mm01149710_m1
Nitric Oxide Synthase 2	*Nos2*	Mm00440502_m1
Pentraxin 3	*Ptx3*	Mm00477268_m1
Peroxisome proliferator-activated receptor ganna	*Pparg*	Mm00440940_m1
TSG6 (TNF-inducible gene 6 protein)	*Tnfaip6*	Mm00493736_m1
Versican	*Vcan*	Mm01283063_m1

(a)

	1	2	3	4	5	6	7	8	9	10	11	12
**A**	18s rRNA Hs99999901_s1	Btk Mm00442712_m1	Casp8 Mm00802247_m1	Ccl2 Mm00441242_m1	Cd14 Mm00438094_g1	Cd80 Mm00711660_m1	Cd86 Mm00444543_m1	Cebpb Mm00843434_s1	Chuk Mm00432529_m1	Clec4e Mm00490870_m1	Csf2 Mm01290062_m1	Csf3 Mm00438334_m1
**B**	Cxcl10 Mm00445235_m1	Elk1 Mm00468230_m1	Fadd Mm00438861_m1	Fos Mm00487425_m1	Hmgb1 Mm00849805_gH	Hras Mm01275929_m1	Agfg1 Mm00802809_m1	Hspa1a Mm01159846_s1	Hspd1 Mm00849835_g1	Ifnb1 Mm00439552_s1	Ifng Mm01168134_m1	Ikbkb Mm01222247_m1
**C**	Il10 Mm00439614_m1	Il12a Mm00434165_m1	Il1a Mm00439620_m1	Il1b Mm01336189_m1	Il1r1 Mm00434237_m1	Il2 Mm00434256_m1	Il6 Mm00446190_m1	Il6ra Mm00439653_m1	Irak1 Mm01193538_m1	Irak2 Mm01184677_m1	Irf1 Mm01288580_m1	Irf3 Mm00516779_m1
**D**	Jun Mm00495062_s1	Lta Mm00440228_gH	Muc13 Mm00495397_m1	Ly86 Mm00440240_m1	Ly96 Mm01227593_m1	Map2k3 Mm00435950_m1	Map2k4 Mm00436508_m1	Map3k1 Mm00803707_m1	Map3k7 Mm00554516_g1	Mapk8 Mm00489514_m1	Mapk8ip3 Mm01301203_m1	Mapk9 Mm00444239_m1
**E**	Myd88 Mm00440338_m1	Nfkb1 Mm00476361_m1	Nfkb2 Mm00479807_m1	Nfkbia Mm00477798_m1	Nfkbib Mm00456849_m1	Nfkbil1 Mm00447990_m1	Nfrkb Mm00555264_m1	Nr2c2 Mm01182440_m1	Peli1 Mm00481051_m1	Pglyrp1 Mm00437150_m1	Ppara Mm00440939_m1	Eif2ak2 Mm01235643_m1
**F**	Ptgs2 Mm00478374_m1	Rel Mm01239661_m1	Rela Mm00501346_m1	Ripk2 Mm00446816_m1	Tbk1 Mm00451150_m1	Ticam1 Mm00844508_s1	Ticam2 Mm01260003_m1	Tirap Mm00446502_m1	Tlr1 Mm00446095_m1	Tlr2 Mm00442346_m1	Tlr3 Mm00628112_m1	Tlr4 Mm00445273_m1
**G**	Tlr5 Mm00546288_s1	Tlr6 Mm02529782_s1	Tlr7 Mm00446590_m1	Tlr8 Mm04209873_m1	Tlr9 Mm00446193_m1	Tnf Mm00443258_m1	Tnfaip3 Mm00437121_m1	Tnfrsf1a Mm00441883_g1	Tollip Mm00445841_m1	Tradd Mm01251031_g1	Traf6 Mm00493836_m1	Ube2n Mm00779119_s1
**H**	Ube2v1 Mm01249540_g1	Gapdh Mm99999915_g1	Hprt Mm00446968_m1	Gusb Mm00446953_m1	Actb Mm00607939_s1	B2m Mm00437762_m1	Hmbs Mm00660262_g1	Ipo8 Mm01255158_m1	Pgk1 Mm00435617_m1	Rplp2 Mm00782638_s1	Tbp Mm00446973_m1	Tfrc Mm00441941_m1

(b)

	1	2	3	4	5	6	7	8	9	10	11	12
**A**	18s rRNA Hs99999901_s1	Adipoq Mm00456425_m1	Ager Mm01134790_g1	Anxa1 Mm00440225_m1	Axl Mm00437221_m1	C3 Mm00437838_m1	Calr Mm00482936_m1	Cd14 Mm00438094_g1	Cd36 Mm01135198_m1	Cd44 Mm01277163_m1	Cd47 Mm00495011_m1	Ceacam3 Mm01257219_m1
**B**	Clec7a Mm01183349_m1	Clic4 Mm01179940_mH	Cnn2 Mm01169510_m1	Colec12 Mm01236242_m1	Crk Mm00467065_m1	Crp Mm00432680_g1	Csf1 Mm00432686_m1	Csf2 Mm01290062_m1	Csk Mm00432751_m1	Cyp2s1 Mm00512037_m1	Dock1 Mm01269874_m1	Dock2 Mm00473720_m1
**C**	Elmo1 Mm00519109_m1	Fas Mm01204974_m1	Fcer1g Mm02343757_m1	Fcgr1 Mm00438874_m1	Fcgr2b Mm00438875_m1	Fcgr3 Mm00438882_m1	Fyn Mm00433373_m1	Gulp1 Mm00518428_m1	Ifng Mm01168134_m1	Il1rl1 Mm00516117_m1	Iqsec1 Mm01182566_m1	Itgam Mm00434455_m1
**D**	Itgav Mm00434486_m1	Itgb2 Mm00434513_m1	Lyn Mm01217488_m1	Mapk14 Mm01301009_m1	Marco Mm00440265_m1	Mbl2 Mm00487623_m1	Mcoln3 Mm00460328_m1	Mertk Mm00434920_m1	Mfge8 Mm00500549_m1	Mif Mm03938638_s1	Msn Mm00447889_m1	Myd88 Mm00440338_m1
**E**	Nod1 Mm00805062_m1	Pecam1 Mm01242584_m1	Pik3cb Mm00659576_m1	Pip5k1a Mm00476829_m1	Pla2g4a Mm00447040_m1	Pla2g5 Mm00448162_m1	Pld1 Mm01289339_m1	Pld2 Mm00447066_m1	Prkce Mm00440894_m1	Pros1 Mm01343426_m1	Pten Mm00477208_m1	Rab5a Mm00727887_s1
**F**	Rab7 Mm01183732_g1	Rac1 Mm01201653_mH	Rac2 Mm00485472_m1	Rala Mm00450933_m1	Ralb Mm00469677_m1	Rapgef3 Mm00522941_m1	Rhoa Mm00834507_g1	Scarb1 Mm00450234_m1	Serpine1 Mm00435860_m1	Sftpd Mm00486060_m1	Siglec1 Mm00488332_m1	Sirpb1a Mm02525668_u1
**G**	Stab2 Mm00454684_m1	Stx18 Mm00452814_m1	Syk Mm01333032_m1	Tgm2 Mm00436987_m1	Ticam1 Mm00844508_s1	Tlr3 Mm01207404_m1	Tlr9 Mm00446193_m1	Tnf Mm00443258_m1	Tnfsf11 Mm00441906_m1	Vamp7 Mm00807071_m1	Vav1 Mm01232047_m1	Was Mm00494167_m1
**H**	Wnt5a Mm00437347_m1	Gapdh Mm99999915_g1	Hprt Mm00446968_m1	Gusb Mm00446953_m1	Actb Mm00607939_s1	B2m Mm00437762_m1	Hmbs Mm00660262_g1	Ipo8 Mm01255158_m1	Pgk1 Mm00435617_m1	Rplp2 Mm00782638_s1	Tbp Mm00446973_m1	Tfrc Mm00441941_m1

(c)
